# The Disease Progression and Molecular Defense Response in *Chenopodium Quinoa* Infected with *Peronospora Variabilis*, the Causal Agent of Quinoa Downy Mildew

**DOI:** 10.3390/plants11212946

**Published:** 2022-11-01

**Authors:** Oscar M. Rollano-Peñaloza, Valeria Palma-Encinas, Susanne Widell, Patricia Mollinedo, Allan G. Rasmusson

**Affiliations:** 1 Department of Biology, Lund University, SE-223 62 Lund, Sweden; 2 Instituto de Investigaciones Químicas, Universidad Mayor de San Andrés, La Paz P.O. Box 12958, Bolivia

**Keywords:** downy mildew disease, *Peronospora variabilis*, *Chenopodium quinoa*, gene expression, chlorosis

## Abstract

Downy mildew disease, caused by the biotrophic oomycete *Peronospora variabilis*, is the largest threat to the cultivation of quinoa (*Chenopodium quinoa* Willd.) in the Andean highlands, and occurs worldwide. However, so far, no molecular study of the quinoa–*Peronospora* interaction has been reported. Here, we developed tools to study downy mildew disease in quinoa at the gene expression level. *P. variabilis* was isolated and maintained, allowing the study of downy mildew disease progression in two quinoa cultivars under controlled conditions. Quinoa gene expression changes induced by *P. variabilis* were analyzed by qRT-PCR, for quinoa homologues of *A. thaliana* pathogen-associated genes. Overall, we observed a slower disease progression and higher tolerance in the quinoa cultivar Kurmi than in the cultivar Maniqueña Real. The quinoa orthologs of putative defense genes such as the catalase *CqCAT2* and the endochitinase *CqEP3* showed no changes in gene expression. In contrast, quinoa orthologs of other defense response genes such as the transcription factor *CqWRKY33* and the chaperone *CqHSP90* were significantly induced in plants infected with *P. variabilis*. These genes could be used as defense response markers to select quinoa cultivars that are more tolerant to *P. variabilis* infection.

## 1. Introduction

Quinoa (*Chenopodium quinoa* Willd.) is an allotetraploid annual crop in the amaranth family domesticated by pre-Columbian civilizations in the central Andes of South America approximately 7000 years ago. Quinoa grains have gained increasing importance in the food market because of their high nutritional value [1,2]. The ability of quinoa to endure severe drought and high salt concentrations has further raised the interest in quinoa to meet the planet’s future food demands [1,3,4,5]. However, quinoa production in the major cultivation areas is limited by downy mildew disease, which can reduce the yield by 35–90% [6,7,8,9]. Downy mildew disease is caused by oomycetes of the *Peronosporaceae* family, and particularly in quinoa by *Peronospora variabilis* Gäum. [4,10,11]. *P. variabilis* has been found present in every continent where quinoa is cultivated [12,13,14,15,16] and its worldwide distribution has likely been expanded by commercial trade of infected seeds [17,18].

*P. variabilis* specifically infects *Chenopodium* species and is an obligate biotroph [19,20]. Little is known about *P. variabilis* biology, including mode of transmission, leaf penetration and signals for sporangiospore and oospore formation [21]. Most of the studies of *P. variabilis* have been directly oriented on screening quinoa cultivars for resistance in agricultural fields [6,7,8,16,22,23]. Some studies have evaluated the resistance of different quinoa cultivars to *P. variabilis* infection under controlled conditions [24,25] or detached leaf assays [12], but molecular studies of the interaction of quinoa with *P. variabilis* or downy mildew disease progression are not available. With the recent availability of the genomic sequences of quinoa [26,27,28] and the close relatives to *P. variabilis, P. tabacina* [29] and *Hyaloperonospora arabidopsidis* [30,31], improved methodology and knowledge on the infection cycle of *P. variabilis* can open up for detailed molecular studies of the quinoa–*Peronospora* pathogenic interaction.

In order to facilitate the study of downy mildew disease in quinoa, we developed a method to isolate and maintain *P. variabilis*. Using this system, we further investigated if there is a variability in susceptibility and mode of infection between cultivars. Hence, we describe downy mildew disease progression in two quinoa cultivars with different tolerance under controlled conditions. The cultivar with higher tolerance to the pathogen attack was selected to identify its defense response mechanisms. The results suggest that quinoa after 48 h of infection with *P. variabilis* expresses the regulatory defense-related genes (*CqWRKY33* and *CqHSP83*).

## 2. Results

### 2.1. Isolation and Characterization of P. variabilis Isolate Kari

**Figure 1:**
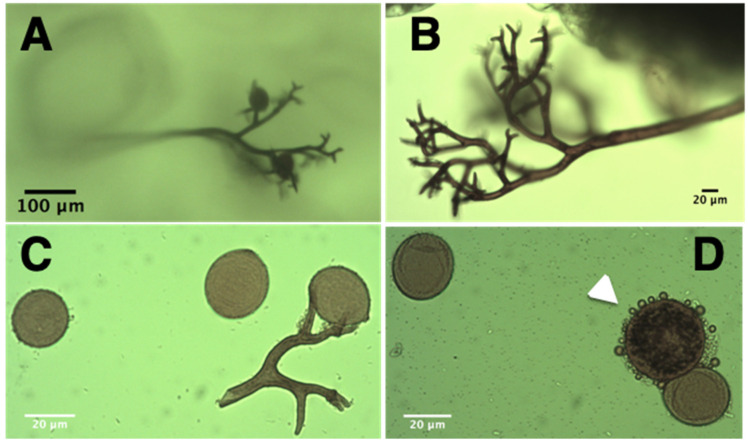
Stained sporangiophores, sporangiospores and oospores of *P. variabilis.* (**A**) sporangiophore branches loaded with sporangiospores growing on the adaxial (top) surface of quinoa leaves; (**B**) sporangiophores growing from quinoa leaves; (**C**) stained sporangiospores of *P. variabilis*, note the broken branch holding sporangia; (**D**) an oospore (white arrow) next to a sporangiospore.

*P. variabilis* was collected from a quinoa greenhouse infected with downy mildew disease. Whole quinoa plants infected were transported to our experimental facility greenhouse for further study. The selected plants displayed main disease symptom by having the leaf abaxial side heavily covered with dark grey sporulation structures (Figure 1). Sporangiospores were collected into sterile water supplemented with the fungicide propiconazole. The addition of the fungicide was essential, as preliminary experiments without it did not lead to infection in consecutive steps. Within 3 h, the sporangiospore suspension was inoculated onto four-week-old quinoa cv. Real plants. These were covered immediately to increase humidity, and thus increase the likelihood of *P. variabilis* infection and sporulation. Seven days post inoculation (dpi) we observed heavy sporulation in the abaxial part of some leaves. The sporangiospores were collected, suspended and inoculated onto leaves of healthy three-week-old quinoa plants. Thereafter, *P. variabilis* was maintained in quinoa cv. Real plants by transfer to new quinoa plants every two weeks under controlled conditions. No infection by *P. variabilis* was observed if propiconazole was omitted from the procedure.

### 2.2. Downy Mildew Disease Progression in Two C. quinoa Cultivars

We evaluated the downy mildew disease progression in two quinoa cultivars upon four repeated infections. Quinoa cultivars Real and Kurmi were selected based on their different susceptibility to *P. variabilis* under field conditions (A. Bonifacio; personal communication). Three-week-old quinoa plants inoculated with a *P. variabilis* sporangiospore suspension started showing folded and moderately chlorotic leaves 5 days after the inoculation (5 dpi), being initial signs of downy-mildew infection. Chlorosis of leaves was obvious in the Kurmi cultivar but was barely observed in the cultivar Real. At 5 dpi none of the cultivars presented sporulation.

**Figure 2:**
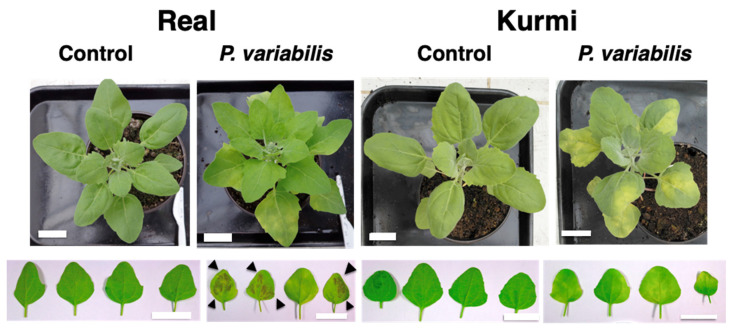
Quinoa plants infected with *P. variabilis*. The figure shows four-week-old quinoa plants 7 dpi with *P. variabilis*. Leaf yellowing is shown from the adaxial side (top) and *Peronospora* sporulation is shown from the abaxial side of freshly detached leaves from the second true leaf pair (bottom). The quinoa cultivar Real displays sporulation (black arrowheads) and the Kurmi cultivar only chlorosis. The images are representatives of at least three independent experiments with three to six biological replicates, all showing similar results. Each leaf in the lower panel was taken from a different biological replicate. The scale bars denote 4 cm.

**Figure 3:**
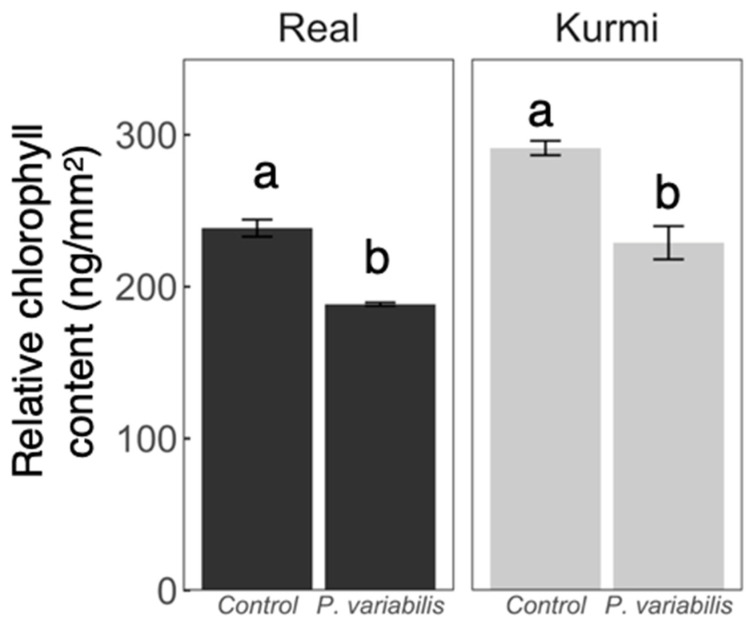
Chlorophyll content in quinoa leaves infected with *P. variabilis*. The second true leaf of three-week-old quinoa plants at 7 dpi was collected and analyzed. Data show means ± SE per treatment (*n* = 4). Different letters mean statistically significant differences according to Student’s t test (*p* ≤ 0.05).

Seven days after inoculation (7 dpi) the Real cultivar started to show sporulation signs on the abaxial side of the leaves, but the Kurmi cultivar did not (Figure 2). At this timepoint chlorosis signs were by eye clearly stronger in cv. Kurmi than in cv. Real (Figure 2). However, image analysis showed that in both quinoa cultivars the chlorophyll content was significantly lower in infected plants as compared to control plants at 7 dpi (Figure 3).

**Figure 4:**
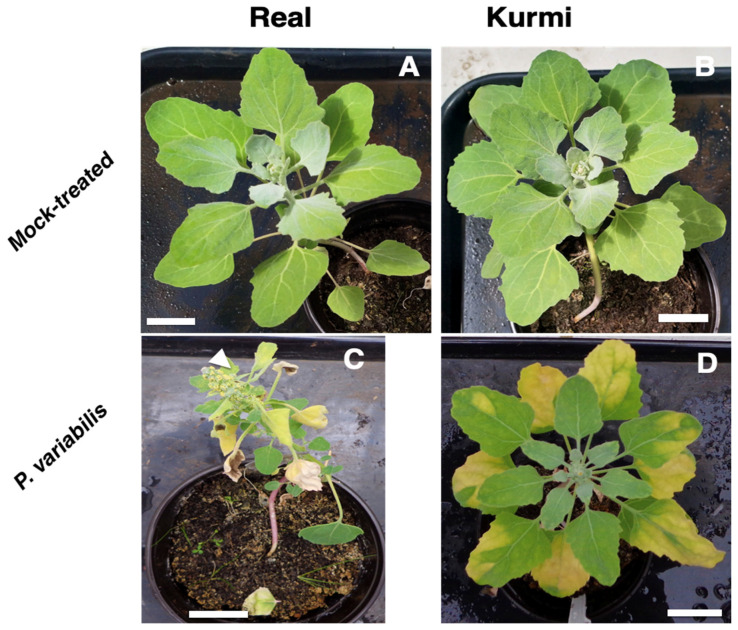
Six-week-old quinoa plants 21 days after infection with *P. variabilis*. The figure shows symptoms of downy mildew disease in quinoa cv. Real (**C**) and cv. Kurmi (**D**), as compared to mock-treated controls (**A** and **B**, respectively). The images are representatives of two independent experiments with three biological replicates each. The white arrowhead points at the early flowering observed in the infected Real cultivar.

The effect on vitality of quinoa was further evaluated until 21 dpi (Figure 4). Here, we observed that at 9 dpi, both the Kurmi and Real cultivars showed heavy sporulation from the abaxial side of the leaves, and some sporulation could be observed on the adaxial side of the leave (Figure 2). Both cultivars grew vividly in the absence of the pathogen over the 21 days. However, plants of cultivar Real infected with *P. variabilis* were severely negatively affected at 21 dpi (Figure 4C) as compared to the mock-treated plants (Figure 4A). The Real cultivar infected with *P. variabilis* showed sporulation in most of its leaves and many of them were wilting or dead at 21 dpi (Figure 4C). In contrast, cultivar Kurmi displayed infected leaves, but the sporulation was localized only to the chlorotic parts of the leave (Figure 4D). In both the Kurmi and Real cultivars, new leaves emerged without signs of infection. However, in cultivar Real the visibly unaffected leaves were mainly derived from side-branches, whereas the unaffected leaves in cultivar Kurmi emerged from the main stem as well as from side-branches. The Real cultivar infected with *P. variabilis* also induced early flowering (Figure 4C) as compared to its mock-treated counterpart (Figure 4B). Early flowering was not observed in the Kurmi cultivar (Figure 4D). Defined necrotic lesions were not observed at any time point.

In general, we observed that the Kurmi cultivar had clearly better defense response mechanisms to resist *P. variabilis* infection than the Real cultivar. Therefore, we selected cultivar Kurmi to perform gene expression analysis to understand further the quinoa defense response.

### 2.3. Quinoa Gene Expression Response against P. variabilis

**Figure 5:**
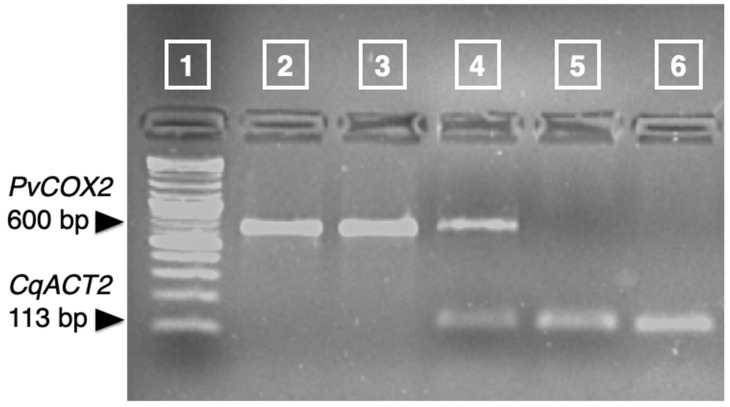
Detection of *P. variabilis* by RT-PCR of the cytochrome *c* oxidase subunit 2 gene. Lane **1**: 2-log DNA ladder (New England Biolabs); lane **2**: *P. variabilis* genomic DNA; lane **3**: *P. variabilis* cDNA; lane **4**: cDNA derived from a plant infected with *P. variabilis*, amplicons of PvCOX2 and CqACT2 of the same sample were pooled down; lane **5**: cDNA derived from a mock-treated plant; lane **6**: *C. quinoa* genomic DNA.

Quinoa plants did not show any infection signs during the first 5 days after inoculation. In order to investigate if the plants are infected at an earlier sampling time suitable for gene expression response analyses (e.g., 2 dpi) we performed molecular detection of *P. variabilis* in leaf RNA samples. RT-PCR of the *P. variabilis* cytochrome *c* oxidase subunit 2 gene (*PvCOX2*) produced a 600 bp product in plants infected with *P. variabilis* at 2 dpi, whereas no PCR product was observed in mock-treated plants (Figure 5). This verified that the treated quinoa plants were infected with *P. variabilis* at the time of sampling.

Quinoa putative reference genes for gene expression analysis were selected based on *Arabidopsis thaliana* microarray data indicating expressional stability under stress [32,33]. We verified the presence of *A. thaliana* gene orthologs in quinoa by two-way BLASTp searches [34] against the quinoa genome. Most of the genes in *A. thaliana* have at least two copies in quinoa, maybe because quinoa is allotetraploid [26]. Therefore, the top hit from each BLAST search was selected for primer design. The gene selected was identified with a letter “A” at the end of the gene name abbreviation and the genes with lower scores were listed in order of sequence similarity to the *A. thaliana* gene (Table 1).

**Figure 6:**
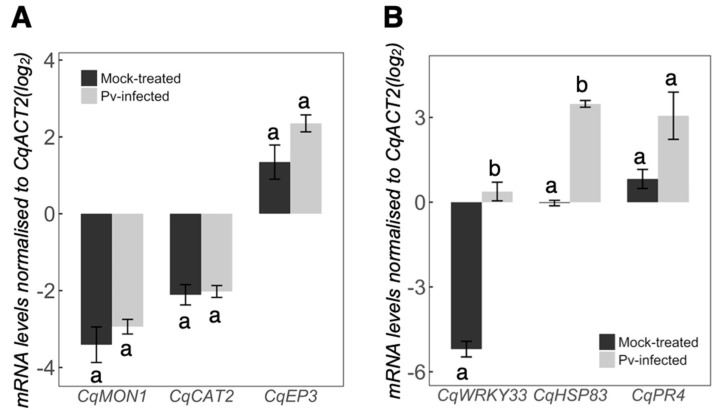
Gene expression in quinoa plants in response to *P. variabilis*. The figure shows defense response gene orthologs of quinoa cv. Kurmi at 48 h after spray-inoculation with *P. variabilis* or mock control. (**A**) Quinoa reference gene (*CqMON1*) and protein-coding genes (*CqCAT2* and *CqEP3*). (**B**) Quinoa genes (*CqHSP83*, *CqWRKY33* and *CqPR4*) with putative defense response functions. Data are shown as average log2 ratios of target gene to *CqACT2*, and error bars denote SE. Significant differences (*p* < 0.05) between infected and mock are denoted by different lowercase letters.

The genes selected as reference genes were the orthologs of *A. thaliana Actin-2 (At3g18780, AtACT2)* and *Monensin Sensitivity 1 (At2g28390, AtMON1)*. *AtACT2* scored 4 ortholog sequences with high similarity, whereof *AUR62014374* displayed the highest BLAST score (*CqACT2A*; denoted in our study as *CqACT2*). *AtMON1* had two quinoa orthologs: *AUR62020295* and *AUR62037705* (identified as *CqMON1A* and *CqMON1B,* respectively). Due to the high nucleotide sequence identity (98%) between the *CqMON1* orthologs, primer pair used targeted both genes. We denote both targeted genes as only one *CqMON1* (Table 1). The putative quinoa reference genes showed similar stability in amplification as it can be appreciated by their average Ct and standard error (*CqACT2* = 20 ± 0.6; *CqMON1* = 23 ± 0.5). *CqACT2* was selected as the reference gene due to its higher expression levels. The results were verified by *CqMON1,* which showed similar expression with and without *P. variabilis* treatment (Figure 6A).

We decided to test known protein-coding genes involved in defense responses of *A. thaliana* to biotrophic infection. Thus, we investigated the quinoa orthologs of *Arabidopsis CATALASE2* (*AtCAT2* [35]) and one chitinase-encoding gene (*AtEP3* [36]). However, we observed no changes in the expression of these genes upon infection with *P. variabilis*.

Given that the studied protein-coding genes were not affected by *P. variabilis* infection, we decided to evaluate the involvement of other quinoa genes with putative functions related to defense response (Table 1). We selected quinoa genes (*CqHSP83*, *CqWRKY33* and *CqPR4*), all of which have been shown to be differentially expressed upon biotic interactions [37]. All these genes displayed elevated mRNA abundance values in plants infected with *P. variabilis* but only *CqHSP83 (p =* 2 × 10^−5^*)* and *CqWRKY33 (p* = 2 × 10^−4^) were significantly different from the mock-treated plants at 48 hpi (Figure 6B).

## 3. Discussion

The novel *P. variabilis* isolate Kari (Figure 1) displayed vegetative and reproductive structures and produced disease symptoms similar to other isolates described before [17,21]. Propiconazole was crucial for successful isolation of the *P. variabilis* Kari strain, most likely because it inhibited the growth of fungi that can parasitize on or compete with oomycetes like *P. variabilis*. Growth inhibition in fungi, but not in oomycetes, is achieved because propiconazole inhibits one of the steps in the synthesis of ergosterol. This is a major sterol in fungi [38], whereas *Peronospora* and other Peronosporales (Oomycetes) do not synthesize ergosterol [39]. Sequencing of the ITS region verified that the Kari strain, isolated from the Bolivian Andean plateau, belongs to the *P. variabilis* species [11,17].

*Peronospora variabilis* isolate Kari was compatible with both investigated quinoa cultivars, Real and Kurmi (Figure 2 and Figure 4). The downy mildew disease symptoms produced by *Peronospora variabilis* isolate Kari were chlorosis, foliar curling, and heavy sporulation on the abaxial side of the leaves. These symptoms are consistent with the downy mildew disease symptoms in susceptible quinoa cultivars, as observed in agricultural fields [9,17].

Growth and development of the Real cultivar was more affected by *P. variabilis* than the Kurmi cultivar (Figure 4). The early flowering in quinoa cv. Real produced by *P. variabilis* is a typical symptom of stress-induced flowering [40]. Stress-induced early flowering has previously been observed in *A. thaliana* infected with the oomycete *H. arabidopsidis* [41]. The early and more pronounced chlorosis in infected plants of the Kurmi cultivar, yet over time better growth than was displayed by infected plants of cultivar Real (Figure 2 and Figure 4), indicate that cultivar Kurmi is more tolerant to *P. variabilis* infection than the Real cultivar, and that the tolerance mechanisms are active after the initial stage of infection. The tolerance of Kurmi is consistent with the high resistance to downy mildew observed in the Kurmi cultivar compared to the Real cultivar in cultivations of quinoa in the Andean plateau as described by A. Bonifacio (personal communication) and previous reports showing the susceptibility of Real cultivar to downy mildew [42]. Therefore, we suggest the cultivar Kurmi is a suitable candidate to study quinoa defense response mechanisms at molecular level.

The quinoa cultivar Kurmi did not show any signs typical of hypersensitive response (HR) (Figure 2 and Figure 4). HR is normally triggered by plants in order to deter biotroph pathogens [43], suggesting that the studied quinoa cultivars are susceptible to downy mildew due to a lack of hypersensitive response induction mechanisms. Instead, chlorosis signs were observed in infected leaves before the pathogen was visibly sporulating from the abaxial side of the leaf in both quinoa cultivars, being visibly stronger in cultivar Kurmi (Figure 2 and Figure 4). Similar results were observed in *A. thaliana* susceptible varieties (compatible interactions) in response to the infection with the biotroph *H. arabidopsidis* [44]. Chlorosis can be a sign of damage, yet also a signal of a defense response which is usually triggered against necrotrophic pathogens [45]. The unusual defense response observed in quinoa against biotrophic pathogens highlights the need to study with more detail the molecular response of quinoa to downy mildew disease, and its efficiency to counteract the pathogen attack.

*AtCAT2* encodes a putative functional catalase [46] that increases hydrogen peroxide levels to eventually trigger HR [35]. As we did not observe HR in our results, it was expected that *CqCAT2* would have unchanged gene expression upon pathogen infection, as was also observed (*CqCAT2*, Figure 6A). Similarly, the chitinase *CaEP3* of *Chenopodium amaranticolor* has been observed to be expressed during HR mediated by cucumber mosaic virus inoculation [36,47] and is induced by elicitors [48]. However, its quinoa ortholog *CqEP3* was not significantly changed during *P. variabilis* infection (Figure 6A). In contrast, the quinoa ortholog *CqWRKY33* of *Arabidopsis* (*AtWRKY33*), which is known to be strongly induced during infection by necrotrophic pathogens such as *Botrytis cinerea* [49,50] was significantly induced upon infection with *P. variabilis* (Figure 6B). *CqWRKY33* induction by *P. variabilis* suggests that genes involved in hypersensitive response were not triggered in any of the quinoa cultivars tested and that quinoa defense response to *P. variabilis* was related to a necrotrophic-pathogen defense response in *A. thaliana*.

The molecular chaperone *AtHSP90* [51,52] and the chitinase *AtPR4* of *A. thaliana* [53] are both involved in defense responses. Further, the quinoa orthologs of these genes (*CqHSP83* and *CqPR4*) were previously shown to be differentially expressed upon fungal interaction [37]. In our results, *CqHSP83* and *CqPR4* were also induced upon treatment with *P. variabilis*, indicating involvement in defense response. *CqPR4* have shown a strong inhibition fungal infection though it, in contrast to what was previously thought, does not possess chitinase activity [54]. This gene has instead been indicated to interact with fungal lectins and thus to inhibit fungal weapons of pathogenesis [54]. Therefore, this gene might also contribute to hinder oomycete growth in quinoa plants.

*AtHSP90* is a molecular chaperone that has been shown to be induced by biotic stress and suggested to modulate plant cell death through R gene-mediated signaling [51,55]. Its ortholog in quinoa, *CqHSP83* may also play a similar role, given that it was differentially expressed upon treatment with *P. variabilis* (Figure 6) and in other biotic interactions [37]. As quinoa plants did not activate cell death mechanisms during the infection, which generally is a common and efficient plant defense against biotrophs, it could be interesting to analyze the downstream components of this signaling cascade to elucidate the molecular components that trigger hypersensitive cell death in *Arabidopsis* but not in quinoa plants.

Quinoa cv. Kurmi gene expression in response to the oomycete *P. variabilis* displayed similarities to the *A. thaliana* Col-0 compatible interaction response to *H. arabidopsidis* Waco9.

*AtHSP90*, *AtWRKY33* and *AtPR4* were differentially expressed in *A. thaliana* plants after 3 days of infection with *H. arabidopsidis* but the genes *AtEP3* and *AtCAT2* were not [31]. Therefore, we must conclude that despite the higher tolerance to *P. variabilis* infection than the Real cultivar, the Kurmi cultivar is a *Peronospora*-compatible and semi-susceptible cultivar.

It is important to note that the degree of *P. variabilis* compatibility reported here for the cultivars Kurmi and Real might change with a different isolate of *P. variabilis*. This compatibility between plants and pathogens is cultivar- and isolate-specific and is well-described in the plant model *Arabidopsis thaliana* interacting with the oomycete *H. arabidopsidis* [56]. Given the high genetic diversity of quinoa that we can find in the Andean highlands [57], we can also expect a high genetic diversity of *P. variabilis*, including different compatibility properties for the different quinoa cultivars.

In conclusion, both quinoa cultivars were susceptible to infection by the novel *P. variabilis* isolate Kari. The infection has stronger effects over the vitality of cultivar Real, leading to a higher proportion of dead leaves, reduced growth and altered morphology as compared to cultivar Kurmi. Furthermore, none of the cultivars presented signs that would suggest that the quinoa cultivars studied can trigger hypersensitive response in response to *P. variabilis* isolate Kari.

Understanding the molecular response and defense mechanisms of the Kurmi cultivar, which presents a higher tolerance to *P. variabilis* infection, can contribute to the development of resistant quinoa cultivars in future breeding programs.

## 4. Materials and Methods

### 4.1. Plant Material and Growth Conditions

Quinoa (*Chenopodium quinoa* Willd.) seeds of the cultivar Maniqueña Real (Real) and Kurmi were kindly supplied by PROINPA (Quipaquipani, La Paz, Bolivia). Plants were regularly grown and maintained in pots in a greenhouse (Cota Cota, La Paz, Bolivia) under natural light (12 h light/12 h darkness) and at a temperature varying between 17 and 25 °C. Plants were watered three times a week.

### 4.2. Peronospora Variabilis Isolation

Quinoa plants infected with *P. variabilis* were collected from the fields of the PROINPA foundation (Quipaquipani, Bolivia). Whole infected plants were transplanted in situ to pots with fresh soil, covered with plastic bags and transported to our greenhouse. After 24 h, a single-lesion infected leaf was detached from one of the infected quinoa plants and sporangiospores were scraped into sterile water (Milli-Q, Merck Millipore, Burlington, Mass.) supplemented with 25 µg/mL propiconazole (Propilac 25 EC, Guayaquil, Ecuador). The sporangiospore concentration was calculated with the 0.04 mm^2^ unit of an improved Neubauer chamber and adjusted to 1 × 10^6^ sporangiospores per mL with sterile water. Within three hours, the suspension was sprayed up to saturation point onto four-week-old quinoa plants cv. Real; this cultivar has previously been shown susceptible to *P. variabilis* [42]. Immediately after spraying, semi-transparent polyethylene plastic covers were placed on top of the plants to increase humidity. The covers were removed after 24 h. After another 5 days of incubation under greenhouse conditions, plants were covered again for 24 h to favor *P. variabilis* sporulation.

### 4.3. Peronospora Variabilis Maintenance

Every two weeks, the sporangiospores of a single-lesion infected quinoa leaf were collected into a suspension and inoculated onto three-week-old quinoa cv. Real plants as described above, yet without adjusting the sporangiospore concentration.

### 4.4. Microscopy of P. variabilis Structures

Staining of hyphae and sporangiospores was performed as described by Koroch, Villani [58], with some modifications. Briefly, quinoa leaves infected with *P. variabilis* were excised in 1-cm^2^ pieces and placed on a microscope slide with the adaxial side facing the slide. Two drops of a solution of I_2_/KI solution (0.5 g I_2_, 1.5 g KI in 25 mL H_2_O) were placed on the abaxial side of the infected leaf, which was incubated at room temperature for 5 min before a cover slip was placed on top. Images were taken with an Optika Vision Pro light microscope (Olympus, Kansas City, MO, USA).

### 4.5. Molecular Identification of P. variabilis

Total DNA was extracted from *P. variabilis* sporangiospore suspensions using the Purelink genomic DNA Kit according to the instructions of the manufacturer (Thermo Scientific, Carlsbad, CA, USA), with the following modifications: Fresh samples were thoroughly ground under liquid nitrogen in a precooled mortar without letting the samples thaw. Then, 600 µL of Purelink Lysis Buffer was added, grinding continued until the samples had thawed, and samples were transferred to 1.5 mL microcentrifuge tubes. DNA was quantified by fluorometry using a Qubit 2.0 Fluorometer (Thermo Scientific, Carlsbad, CA, USA).

PCR of the Internal Transcribed Spacer (ITS) region [59] was done with primer pairs DC6/ITS4 as described [60] using the Phusion High-Fidelity PCR Master Mix (Thermo Scientific, Carlsbad, CA, USA) supplemented with 0.25 µM of each primer. Genomic DNA (20 ng) was used as template in a 20 µL PCR reaction. The PCR program had the following conditions. One cycle of 98 °C for 30 s; 30 cycles of 98 °C for 30 s, 52 °C for 30 s and 72 °C for 60 s); 1 final cycle of 72 °C for 5 min.

PCR products (150 ng) from the ITS region and the cytochrome *c* oxidase subunit 2 (*PvCOX2)* gene of *P. variabilis* were purified with the QIAquick PCR Purification Kit (Qiagen, Hilden, Germany). The purified PCR products were directly sequenced by the Sanger method (Eurofins, Ebersberg, Germany) and confirmed for the complementary strand as described before [59].

### 4.6. Downy Mildew Disease Progression

Three-week-old quinoa plants (cv. Kurmi and Real) were spray-inoculated (to saturation) with either sterile 25 µg/mL propiconazole in sterile water (control) or with a fresh *P. variabilis* sporangiospore suspension [1 × 10^6^ sp/mL] diluted into sterile water and supplemented with propiconazole (to 25 µg/mL). Treated plants were immediately covered with semi-transparent polyethylene plastic bags to raise humidity. The plastic bag covers were left for 24 h. Quinoa leaves were monitored daily for signs of disease, and plants were photographed at 0, 2, 5, 7, 9 and 21 dpi with a digital camera. Leaves were collected at 7 dpi for chlorophyll analysis. The chlorophyll content was estimated from the abaxial side of the second pair of true leaves as described by Liang, Urano [61].

### 4.7. RNA Isolation and cDNA Synthesis

Plant tissue from quinoa cv. Kurmi was sampled 48 h post infection (hpi) for RNA isolation. Each sample consisted of one leaf from the second pair of true leaves cut in half and immediately frozen with liquid nitrogen.

For *P. variabilis* RNA extraction, the sporangiospore/sporangiophore suspensions were prepared by scraping sporangiophores attached to *C. quinoa* leaves from 9 dpi-infected plants. The sporangiospore/sporangiophore suspension was immediately shock-frozen in liquid nitrogen.

Total RNA from quinoa or *P. variabilis* was extracted using the Purelink RNA Mini Kit (Thermo Scientific, Carlsbad, CA, USA). Briefly, fresh samples were ground under liquid nitrogen in a precooled mortar without letting the samples thaw followed by addition of 1000 µL of Purelink lysis buffer (Thermo Scientific, Carlsbad, CA, USA) supplemented with 2-mercaptoethanol [10 µL/mL]. Grinding continued until samples had thawed, and samples were placed in 1.5 mL microcentrifuge tubes. Thereafter, the RNA extraction was performed as described by the manufacturer.

Isolated RNA was quantified by fluorometry using a Qubit 2.0 and RNA quality was verified by examination of ribosomal RNA bands on agarose gels. Synthesis of cDNA was carried out with 500 ng of total RNA added to each reaction of the High-Capacity cDNA Reverse Transcription Kit (Thermo Scientific, Carlsbad, CA, USA). The cDNA samples were stored at −20 °C for downstream analysis.

### 4.8. Molecular Detection of P. variabilis PvCOX2

RT-PCR of the *PvCOX2* was done with the primer pair previously described by Hudspeth [62] using the Hot Firepol EvaGreen qPCR Mix Plus (Solis BioDyne, Tartu, Estonia) supplemented with 0.25 µM of each primer. Template was 4 µL of cDNA in a final PCR reaction volume of 20 µL. The PCR program was performed in a LifePro thermocycler (Bioer, Hangzhou, China) and had the following conditions: One cycle of 95 °C for 15 min; 30 cycles of 95 °C for 30 s, 50 °C for 30 s and 72 °C for 60 s); 1 final cycle of 72 °C for 5 min. Singularity of PCR products was verified on 2% agarose gels stained with SYBR Safe gel stain (Thermo Scientific, Carlsbad, CA, USA).

### 4.9. Gene Expression Analysis

Plant RNA was analyzed by qRT-PCR in a StepOnePlus Real-Time PCR system (Thermo Scientific, Carlsbad, CA, USA) using Fast SYBR Green Master Mix (Thermo Scientific, Carlsbad, CA, USA) supplemented with 0.25 µM of each specific primer and using cDNA corresponding to 10 ng of isolated RNA as template. The PCR program had the following conditions: 1 cycle of: 95 °C, 20 s; 30 cycles of: (95 °C, 15 s; 60 °C, 20s; 72 °C, 20 s). The specificity of each PCR amplification was determined by melt curve analysis and by electrophoretic analysis in 2% agarose gels. The primer sequences can be found in Table 1. The relative transcript expression was calculated by the Pfaffl algorithm using *CqACT2* and *CqMON1*, as reference genes. Ten-fold dilutions of cDNA template were used to determine the amplification efficiency for each gene [63].

Primer pairs were designed using Perlprimer [64] so that one of the primers in each pair spanned an exon–exon border, and the primer pairs were checked using Netprimer (premierbiosoft.com accessed on 3 October 2015) to avoid primer–primer interactions.

### 4.10. Statistics

Gene expression levels in plants inoculated with *P. variabilis* or mock-treated were compared using Students *t*-test for a significant p-value limit of *p* < 0.05. The statistical analysis was carried out in RStudio (v. 1.0.143), using the R packages plyr [65] and stats [66]. Images were produced using ggplot2 [67].

## Figures and Tables

**Table 1:** *C. quinoa* genes with their respective *A. thaliana* ortholog-codes and primer sequences for qRT-PCR analysis.

*A. Thaliana* Gene Code	*C. Quinoa* Gene	Molecular Function	*C. Quinoa* Gene Code ^a^	Primer Match ^b^ %		Primer Sequence	Product Size	PCR eff. ^c^
				Fw	Rv			
*AT3G18780* *AtACT2*	*CqACT2A*	Structural, *Reference gene*	*AUR62014374*	100	100	‘5-TACCACAGGTATCGTGCTTGACTC-3’ ‘5-GATCACGTCCGGCAAGATCC-3’	113 bp	1.975
	*CqACT2B*		*AUR62019116*	92	100			
	*CqACT2C*		*AUR62014579*	54	33			
	*CqACT2D*		*AUR62039382*	50	29			
*AT2G28390* *AtMON1*	*CqMON1A*	Vacuolar fusion protein, *Reference gene*	*AUR62020295*	100	100	‘5-AAGGATCATCTGACCATAAAGC-3’	145 bp	2.057
	*CqMON1B*		*AUR62037705*	100	100	‘5-TCGTGTCAAGTTAGTTCGGG-3’		
*AT2G38470* *AtWRKY33*	*CqWRKY33A*	Transcription factor	*AUR62006298*	100	100	‘5-TCCTTTACACCTGAGACATCCT-3’	126 bp	1.953
	*CqWRKY33B*		*AUR62026343*	95	96	‘5-ACTGTTCTGTTACCATACCCTGAC-3’		
*AT5G52640* *AtHSP90*	*CqHSP83A*	Molecular chaperone	*AUR62031424*	100	100	‘5-ATTCGGTGTTGGTTTCTACTC-3’	199 bp	1.917
	*CqHSP83B*		*AUR62021118*	81	100	‘5-CCAAGTATTCCAACTGATCTTCC-3’		
*AT3G04720* *AtPR4*	*CqPR4A*	Fungal growth inhibitor	*AUR62004957*	100	100	‘5-GGCAACGTACAATAACTATAACCC-3’ ‘5-TGCCATGTTTGTTACCCTGAG-3’	192 bp	2.017
	*CqPR4B*		*AUR62001001*	100	71			
	*CqPR4C*		*AUR62004958*	100	48			
*AT3G54420* *AtEP3*	*CqEP3A*	Chitinase	*AUR62031317*	100	100	‘5-CCTTCTTTGCTCATGTCACCC-3’ ‘5-CTGCTCCATAGTTGTAGTTCCA-3’	164 bp	1.969
	*CqEP3B*		*AUR62031318*	100	100			
	*CqEP3C*		*AUR62027403*	91	100			
	*CqEP3D*		*AUR62031322*	91	100			
*AT4G35090* *AtCAT2*	*CqCAT2A*	Catalase	*AUR62040809*	100	100	‘5-CCAGGAGTGAGATATAGATCATGGG-3’	145 bp	1.995
	*CqCAT2B*		*AUR62036848*	100	91	‘5-CCCAAAGATTTATCCGCCTGAG-3’		

^a^ Annotation from QQ74 coastal quinoa genome (Jarvis et al. 2017; http://www.cbrc.kaust.edu.sa/chenopodiumdb/ accessed on 31 May 2021). All quinoa genes denoted here produced the corresponding *A. thaliana* gene as top hit upon a BLASTp search against the *A. thaliana* genome. ^b^ Nucleotide sequence identity of primers compared to different genes. ^c^ PCR amplification efficiency calculated according to Pfaffl, 2001. Abbreviations: Fw, Forward primer; Rv, Reverse primer.

## Data Availability

Data are contained within the article.
